# Experimental study on moisture and heat migration and deformation properties of unsaturated soil column under a temperature gradient during rainfall infiltration

**DOI:** 10.1371/journal.pone.0286973

**Published:** 2023-06-23

**Authors:** Chong Zhang, Enlong Liu

**Affiliations:** 1 Institute for Disaster Management and Reconstruction, Sichuan University, Chengdu, Sichuan, China; 2 State Key Laboratory of Hydraulics and Mountain River Engineering, College of Water Resource and Hydropower, Sichuan University, Chengdu, Sichuan, China; Al Mansour University College-Baghdad-Iraq, IRAQ

## Abstract

Mountainous areas in southwest China are rainy in summer. The rainfall infiltration process involves complex soil thermal-hydraulic-mechanical (THM) coupling problems. The researches on soil THM coupling are mostly focused on numerical simulations, whereas the corresponding model tests are relatively few, and the existing model test studies often ignore the effect of temperature gradients in the soil. However, temperature gradients in the soil can cause water migration and affect the THM behavior of soil, so it cannot be ignored. This paper describes an experimental device that can test the changes of temperature, moisture and displacement in unsaturated soil columns with temperature gradients under rainfall infiltration conditions. By using the apparatus, the model tests of homogeneous soil column (H), homogeneous soil column with infiltration (HI), and preferential flow soil column with infiltration (P) under different temperature gradients are respectively conducted, and the results of moisture and heat migration and deformation properties in soils under different conditions are presented and discussed. A rainfall of low intensity and long duration is applied in the experiments, and the temperature of infiltration rainwater is consistent with that of the soil upper boundary. The results show that: (1) The infiltration of rainfall will increase the temperature of the soil column. The appearance of preferential flow results in faster heat transfer within the soil column, but causes the steady-state temperature to be lower than that of the homogeneous soil (HI); (2) Under infiltration conditions, the preferential flow soil column has an earlier outflow time but a later time for water field to reach steady state, while its water distribution is different from that of the homogeneous soils, with accumulation occurring near the end of preferential flow channel; (3) Under the action of temperature gradient, water migration occurs in homogeneous soil column (H), accompanied by soil settlement, while the infiltrated columns (HI and P) exhibit an increase in both water content and top displacement. In addition, the larger the temperature gradient, the more obvious the thermally induced hydraulic-mechanical response. The research results in this paper can provide experimental evidence for the theoretical study and numerical simulation of the soil THM coupling problems.

## 1. Introduction

Mountainous areas in southwest China are rainy and have a greater temperature change in summer, which are easy to cause geological disasters [1, 2]. These phenomena involve the combined effects of temperature gradient, rainfall infiltration and deformation of the soil, which are complex thermo-hydro-mechanical coupling problems. Temperature gradients are generated in the soil under the action of solar radiation [3–7], which will induce moisture migration and affect thermo-hydro-mechanical behavior of the soil [8–12]. While rainfall infiltration will change the moisture distribution in soil, leading to the reduction of soil strength and even soil instability [13, 14]. Moreover, unsaturated soils with macropores are widely distributed under natural conditions [15–17]. Therefore, based on the practical environmental situation, it is necessary to carry out the study on the thermo-hydro-mechanical coupling of unsaturated soils considering temperature gradient and rainfall infiltration, which can help in predicting the slope stability of these areas.

Temperature gradients are widespread in soils, which can cause water movement and mechanical response, which has been investigated by laboratory tests and numerical simulation. Zhang et al. [18] conducted an experimental study of water migration in soil under the effect of temperature gradient by heating one end of the soil column, but the experimental data were not accurate due to the variation of external temperature. Li [19] adopted a frost heave instrument to accurately control the temperature at both ends of the soil column, and quantified the response of liquid water and water vapor to temperature variation in the process of water migration. While Hedayati et al. [20, 21] investigated the moisture and heat transfer in soil columns under different temperature gradients. Besides the data of temperature and moisture content, the thermal properties and heat flux of the soil column were also measured in the tests, which further revealed the mechanism of moisture and heat transfer under one-dimensional conditions. In addition, temperature gradient can also cause displacement changes, such as frost heave and thaw settlement, which are mostly reflected in the study of frozen soil. Similarly, one-dimensional soil column model tests are widely used in the research of this field [22–24]. For the above changes induced by the temperature gradient, different theoretical models have been used to simulate the experimental results, which can well explain the interaction between temperature, moisture and mechanical field. In 1957, Philip and De Vries [25] first established the hydro-thermal coupling model, which has become one of the most widely used models. Afterwards, other scholars [26–28] improved this model so that it could be applied to more complex engineering situations. Additionally, Thomas [29, 30] proposed a three-phase model with full coupling of heat-moisture-gas-mechanics for unsaturated soils, which has been extended and applied to environmental engineering in recent years [31]. On the basis of previous studies, other scholars [32–34] established different theoretical models by fully considering temperature-induced changes in the hydraulic-mechanical parameters of the soil and the coupling effects between the thermo-hydro-mechanical fields. Therefore, the importance of temperature gradient cannot be ignored in the field of geotechnical engineering.

Frequent rainfall in summer will lead to frequent geological disasters. The research on rainfall infiltration includes experiments and numerical simulations, wherein the experimental studies are essentially conducted by indoor soil column model tests. With a self-made large soil column apparatus, Yang [35, 36] systematically investigated the effects of rainfall intensity, duration, runoff, and homogeneity on the water movement of the soil during infiltration. There are different simplified models for water movement during rainfall infiltration according to different boundary conditions, most of which are concentrated in the one-dimensional field and have been widely verified in practice [37–40]. During rainfall, seepage fields are formed inside the unsaturated soil, resulting in soil skeleton compression and changes in pore structure, which eventually lead to soil deformation or instability. Therefore, the rainfall infiltration process could be regarded as a fluid-solid coupling problem. Researchers carried out a large number of theoretical studies and numerical simulations, which provide data and technical support for the optimal design of slopes, stability analysis, and landslide prediction [13, 14, 41, 42]. Under natural conditions, especially the slope in mountainous areas, due to the random distribution of cracks and macropores, the water movement in soil preferentially selects the path that can move quickly, i.e., the phenomenon of preferential flow occurs [15, 16, 43, 44]. As a typical preferential flow, macropore flow has received a lot of attention [45–48]. At present, there is no unified quantitative description for the definition of macropores, but it can be found that the diameter of all macropores is several orders of magnitude larger than that of the soil matrix [49]. The characteristics of water movement in soils with preferential flow are necessarily different from those in homogeneous soils, so these differences must be taken into account when studying the coupling problem during rainfall infiltration.

In the study of rainfall infiltration, the temperature gradient in soil is usually ignored. However, in some cases there may be both temperature gradient and rainfall infiltration occurring in the soil, while the relevant indoor model tests have not been seen yet. In this study, a set of test equipment was developed that can simultaneously consider the temperature gradient of the soil column, the macropore structure, rainfall infiltration and the temperature of rainwater. Then laboratory model tests of homogeneous soil column (H), homogeneous soil column with infiltration (HI), and preferential flow soil column with infiltration (P) under different temperature gradients were performed respectively, and changes in temperature, water content and top displacement under different experimental conditions as well as their interaction are presented and analyzed.

## 2. Experimental setup and procedure

### 2.1 Experimental device

**Fig 1:**
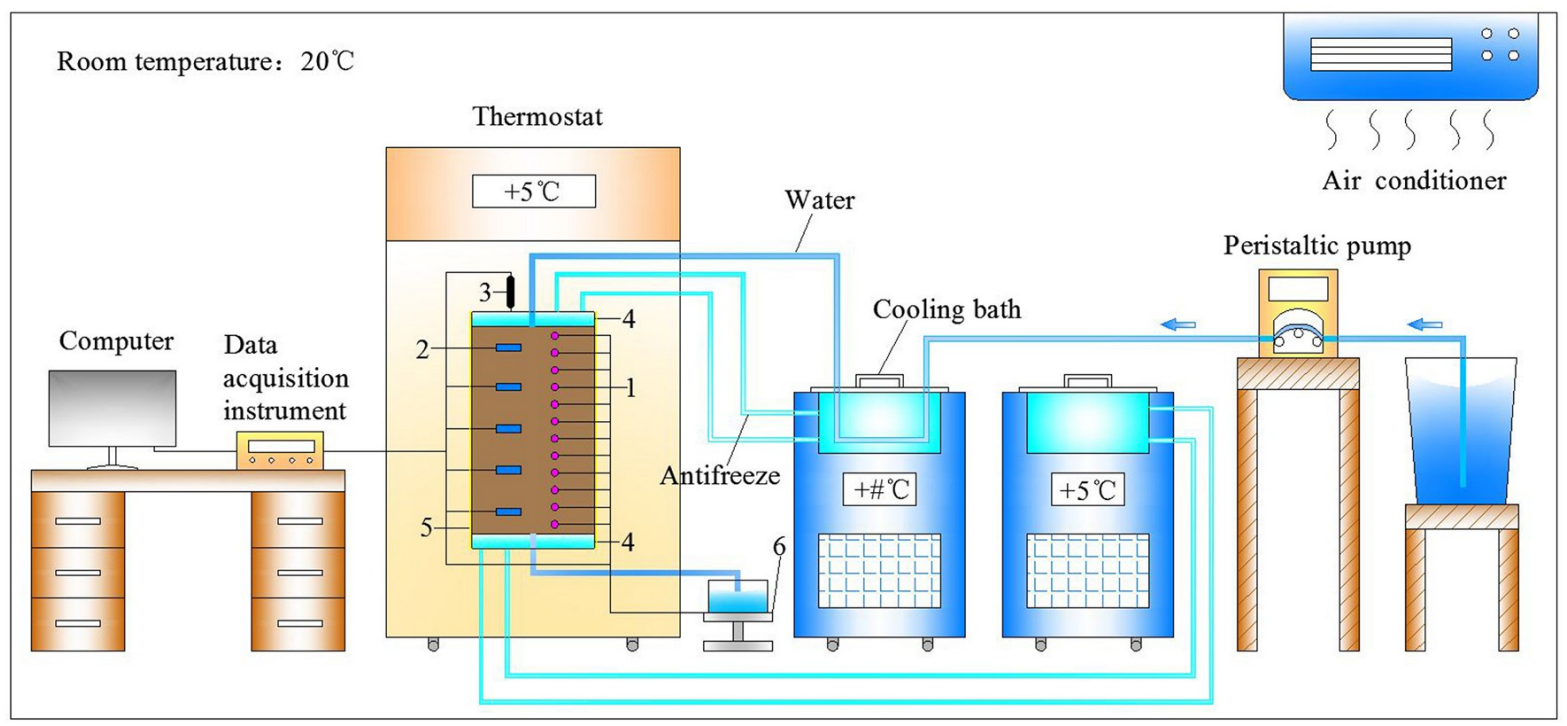
Schematic diagram of the experimental device. (1. temperature sensors, 2. volumetric water content sensors, 3. displacement sensor, 4. metal block, 5. soil column, 6. weighing sensor).

**Fig 2:**
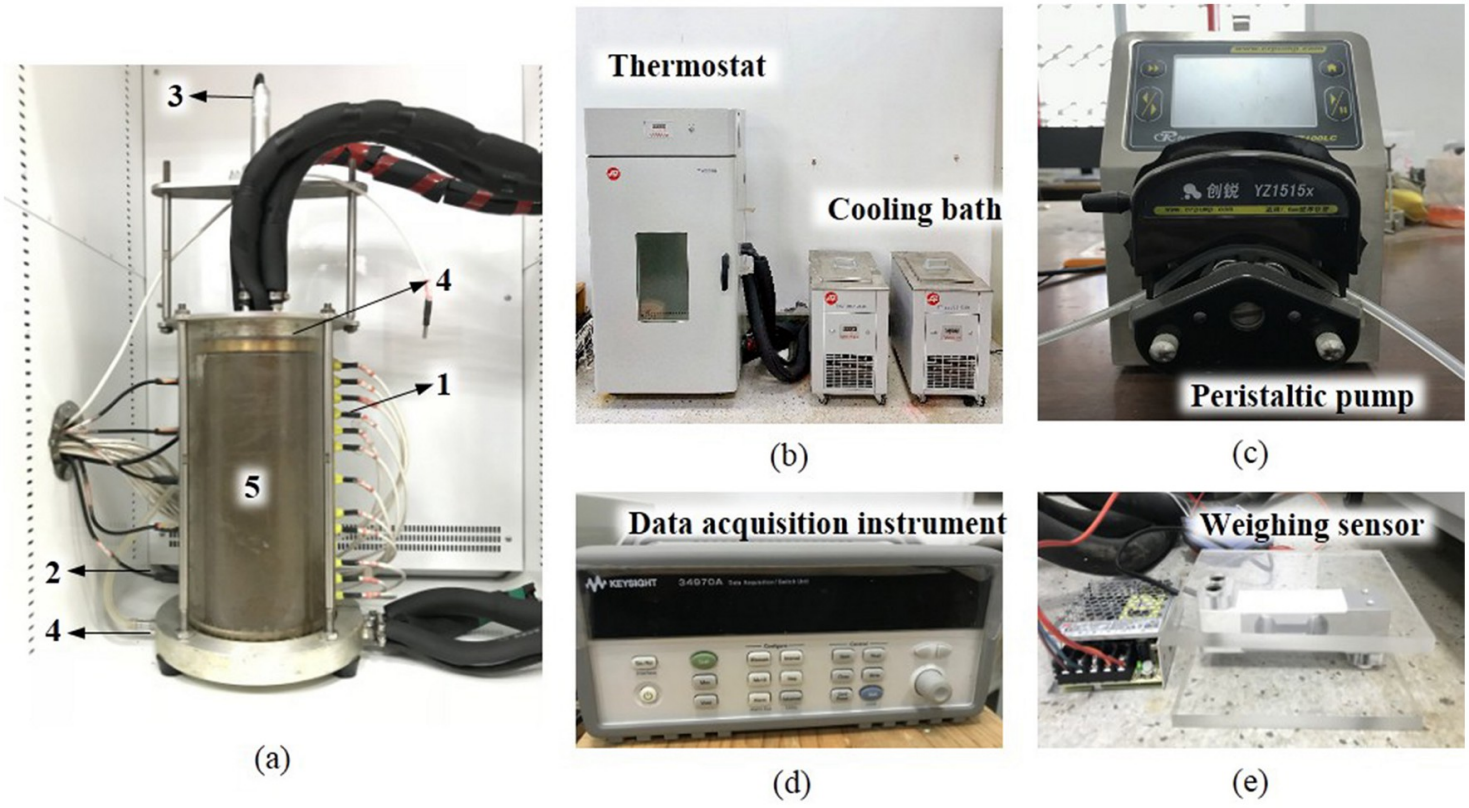
Physical photograph of the experimental device. (a) plexiglass cylinder, (b) temperature control equipment, (c) peristaltic pump, (d) data acquisition instrument, (e) weighing sensor.

Figs 1 and 2 exhibit the schematic diagram and physical photograph of the experimental device, including the temperature control equipment (cooling bath and thermostat), plexiglass cylinder, water supply equipment (peristaltic pump) and water collection equipment, sensors and data acquisition instrument. The metal blocks, which have internal groove channels for liquid flow, are connected to the cooling bath through the pipes. By circulating the antifreeze, the top and bottom metal blocks are uniformly heated to provide stable boundary temperatures for the soil column. The soil column is placed in the thermostat during the test, which ensures a constant ambient temperature of 5 °C. The temperature control range of both the cooling bath and the thermostat is -30 °C to +70 °C with an accuracy of ±0.1 °C.

The plexiglass cylinder has an internal diameter of 150 mm, a wall thickness of 15 mm and a height of 400 mm. As shown in Fig 2(a), the plexiglass cylinder and the stainless-steel ring placed on it, as well as the metal block at its bottom, are assembled together by four metal rods to hold the soil column to be tested and to constrain its bottom and radial movement. During the test, the soil column and its upper metal block are located inside the plexiglass cylinder, and the top of the column can move freely in the vertical direction. Simultaneously, the Vaseline will be evenly applied to the inner wall of the cylinder, which not only reduces the friction at the contact surface between the soil column and the cylinder, but also makes good contact and avoids the flow of water along the cylinder wall. As shown in Fig 2(a), the left side of the cylinder is provided with 5 rectangular holes for the installation of moisture sensors with a spacing of 60 mm, and the right side is provided with 15 circular holes for the installation of temperature sensors with a spacing of 20 mm. In order to effectively prevent water leakage during the test, the joints between the sensors and the cylinder, and the joints between the cylinder and the bottom metal block are sealed with glass glue. Additionally, all the pipes through which the liquid flows, as well as the outside of the plexiglass cylinder, are wrapped with insulation materials to prevent heat loss.

**Fig 3:**
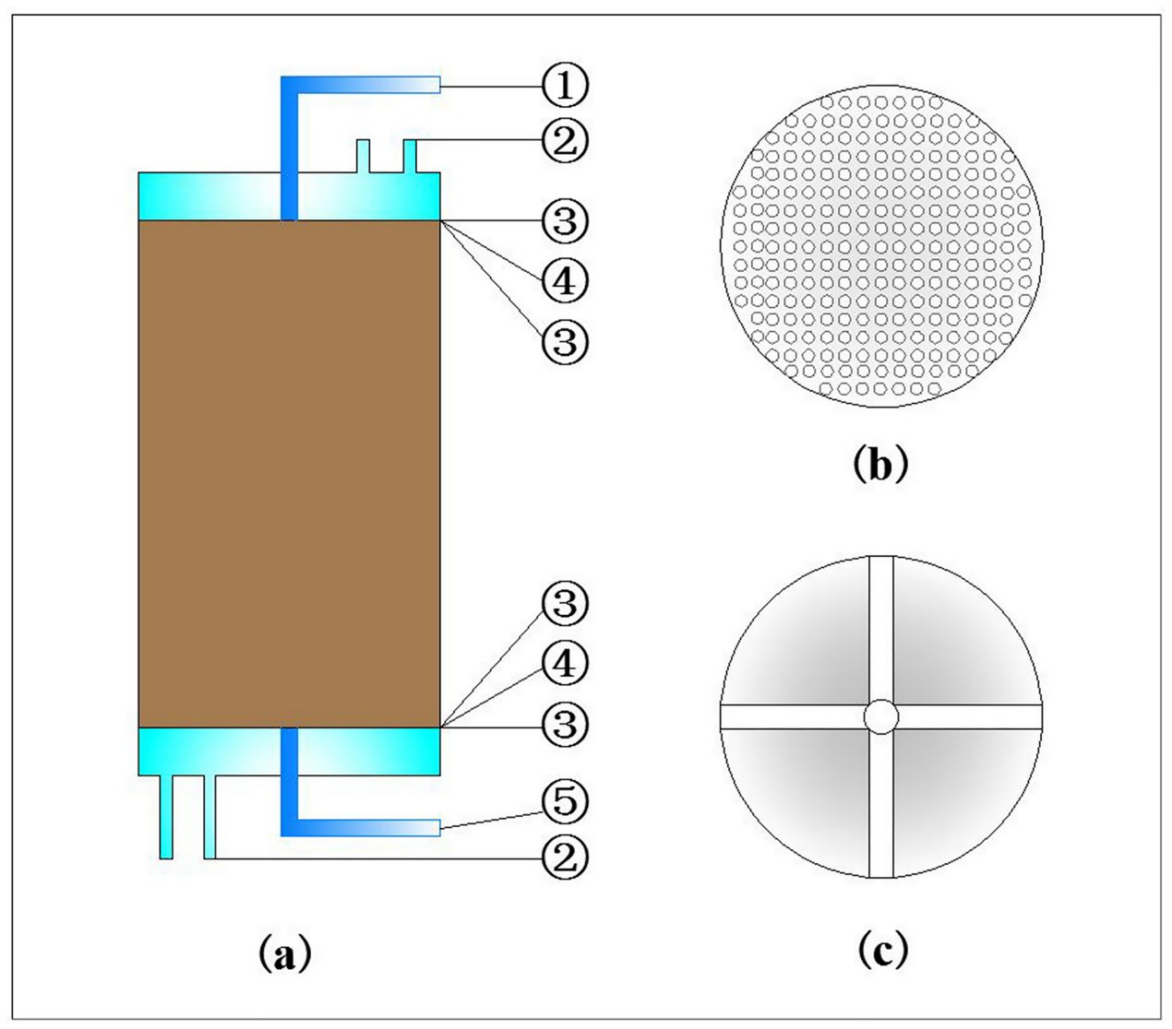
Schematic diagram of some details. (a) soil column: ①infiltration channel, ②antifreeze circulation channel, ③filter paper, ④porous metal sheet, ⑤outflow channel; (b) porous metal sheet; (c) cross recess.

The infiltration channel is located at the center of the metal block at the top of the soil column, with an inner diameter of 6mm. Through a plastic hose connected to the infiltration channel in the metal block, water from the tank is pumped by a peristaltic pump to the surface of the soil column, and a constant infiltration flow is provided. The flow rate used in the study will be calibrated before each infiltration test. To ensure the uniformity of rainfall distribution, filter paper, porous metal sheet, and filter paper are placed sequentially between the top surface of the soil column and the top metal block, as shown in Fig 3. The moisture content at different locations on the surface of the soil column was obtained by the oven drying method, and the comparison of the results showed that the rainfall distribution was essentially uniform in the soil column. According to the relevant research [50, 51], the difference between rainwater temperature and near-surface air temperature is about 1°C in humid and hot areas, while the near-surface air temperature is also relatively close to the surface temperature. Therefore, it is assumed that the infiltration water temperature is the same as the upper boundary temperature of the soil column in this study. As shown in Fig 1, the plastic hose through which the rainwater flows are partially immersed in the cold bath that provides the boundary temperature for the top of the soil column. By adjusting the length of the heated plastic hose, the rainwater temperature will be consistent with the upper boundary temperature of the soil column.

The outflow channel, which has an inner diameter of 6 mm, is located at the metal block at the bottom of the soil column. The percolated water flows out through a channel formed by a hole in the center of the metal block surface and another hole on its right side, and then discharges to the outside of the thermostat through a pipe connected to the block. Meanwhile, a cross recess is set on the surface of the bottom metal block to prevent water from accumulating at the bottom of the soil column, as shown in Fig 3. Similarly, filter paper, porous metal sheet, and filter paper are placed sequentially between the bottom surface of the soil column and the bottom metal block, which served as a filter and support. The plastic container and the weighing sensor are then used to collect and record the weight of the percolated water.

Due to the limited number of data acquisition channels, only 12 temperature sensors with an accuracy of ±0.01 °C are used in this paper. Moreover, the other 3 unused holes at the distance of 0.07 m, 0.13 m, and 0.17 m from the bottom of the soil column are sealed to effectively prevent water leakage. The volumetric water content at different heights are measured by 5 EC-5 moisture sensors with an accuracy of ±0.001 m^3^/m^3^. The displacement sensor with an accuracy of ±0.001 mm is fixed to the metal block at the top of the soil column with a metal bracket. In addition, the data acquisition instrument is connected to the computer to collect and record various data during the experiment, and the data is collected every 1 minute.

### 2.2 Experimental procedure

**Fig 4:**
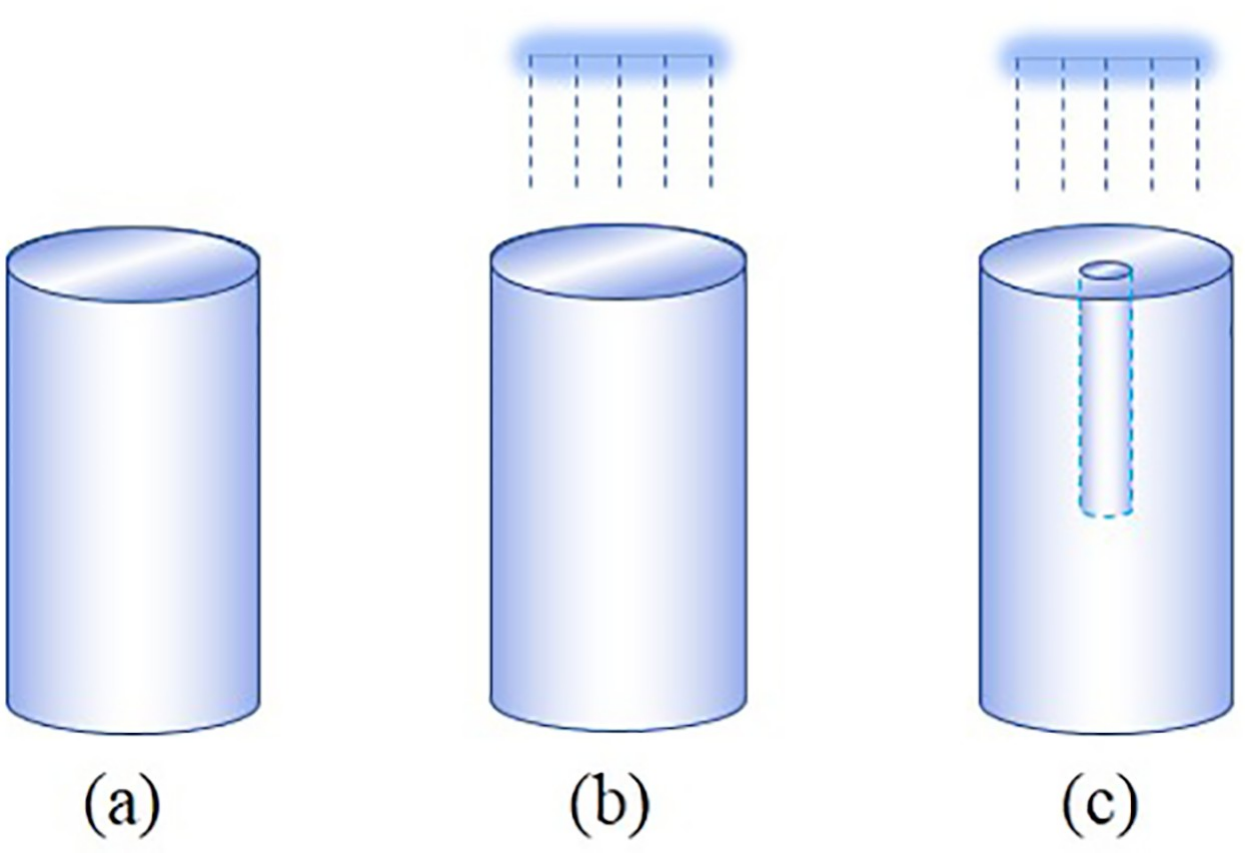
Schematic diagram of the soil column. (a) H, (b) HI, (c) P.

**Fig 5:**
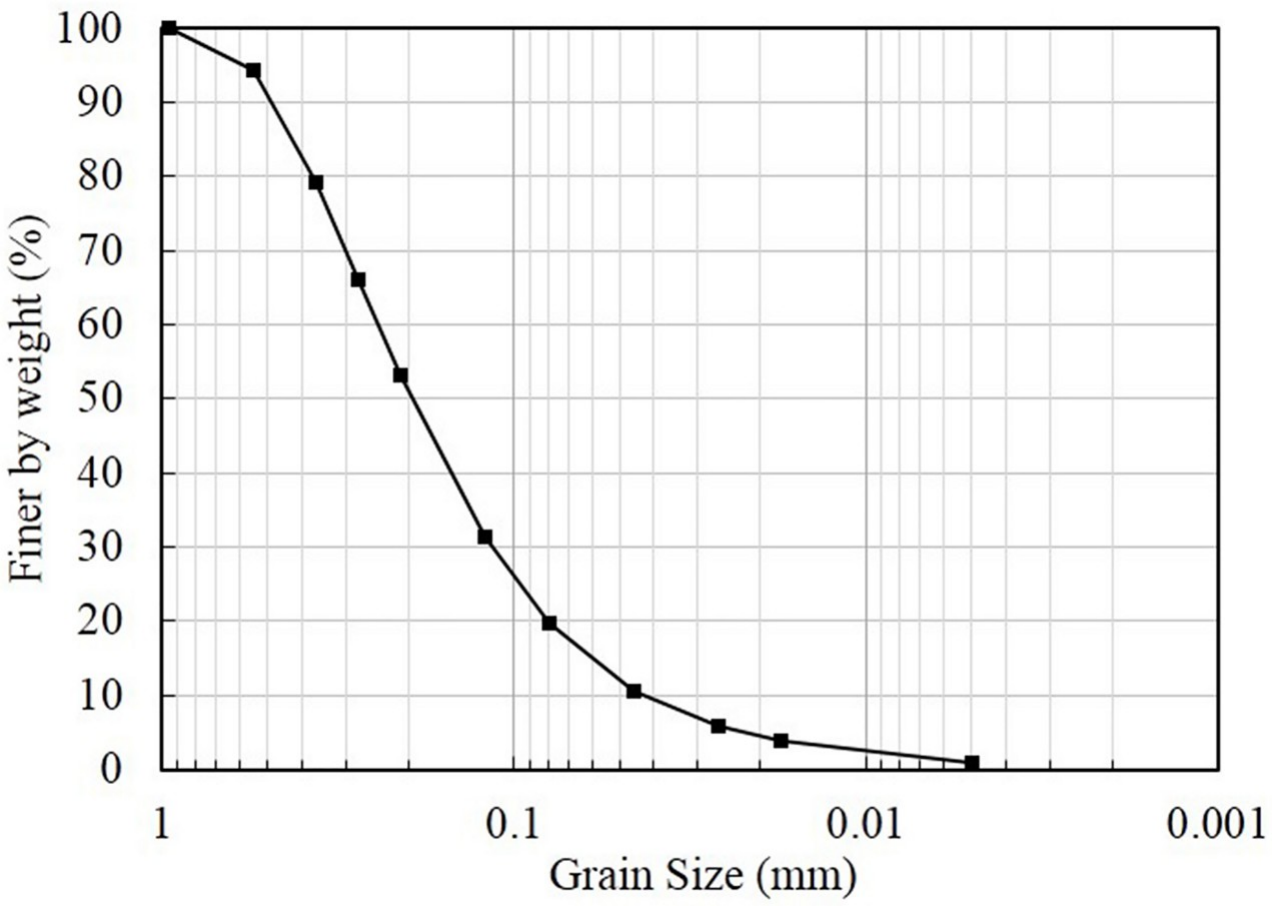
Grain size distribution.

As shown in Table 1, the thermal-hydraulic-mechanical coupled model tests of homogeneous soil column (H), homogeneous soil column with infiltration (HI), and preferential flow soil column with infiltration (P) under different temperature gradients are respectively conducted in this paper. Fig 4 shows the schematic diagram of the soil column. The grain size distribution of the soil used in this paper is shown in Fig 5, which is classified as silty sand by the Unified Soil Classification System. As shown in Table 2, the soil column used in the test has a diameter of 150 mm, a height of 300mm, a dry density of 1.4g/cm^3^, and an initial volumetric water content of 34.3%. While the preferential flow channel, which has a diameter of 20 mm and a height of 150 mm, is located at the center of the soil column and connected to the top of the soil column. Simultaneously, coarse sand with a diameter of 2 mm is evenly filled inside the channel.

**Table 1:** Experimental conditions.

Soil type	Boundary temperature (°C)	
	Top	Bottom
Homogeneous soil column (H)	5	5
	20	5
	27	5
	35	5
Homogeneous soil column with infiltration (HI)	5	5
	20	5
	35	5
Preferential flow soil column with infiltration (P)	5	5
	20	5
	35	5

**Table 2:** Experimental parameters.

Column size (h mm × *φ* mm)	Preferential flow channel size (h mm × *φ* mm)	Dry density (kg/m^3^)	Initial water content (m^3^/m^3^)	Experiment time (h)	Initial temperature (°C)	Infiltration rate (mm/h)		Remark
						H	HI/P	
300 × 100	150 × 20	1.4	0.343	120	5	-	4.1	Free drainage at the bottom

The soil columns used in the test are made by the layered compaction method. However, there are some differences in the sample preparation of the preferential flow soil column. A stainless steel wire mesh was rolled into a hollow cylinder with a diameter of 20 mm to simulate the preferential flow channel. During the sample preparation, the hollow cylinder is placed at the center of the soil column and evenly filled with coarse sand with a diameter of 2 mm. After sample preparation, the hollow cylinder will be removed. By this method [52], the soil columns obtained will hardly be damaged and have good contact between the macroporous areas and homogeneous areas, which are more similar to natural conditions.

After the soil column is prepared, it will be connected to the test device as described above and ready to start the test. The soil column will be kept at a constant temperature of 5 °C for 24 h before the test. By doing this, it can be found the measured data (temperature, water content, displacement) are almost unchanged, which indicates that the thermal-hydraulic-mechanical coupling of the soil column does not change under the action of gravity only. The duration of each test is 120 h, and the infiltration rate is 4.1 mm/h when considering infiltration. The temperature at the lower boundary of the soil column is kept constant during the test, and different temperature gradient conditions are formed by changing the temperature at the upper boundary. Meanwhile, the bottom of soil column can be drained freely in each test.

## 3. Results and discussions

### 3.1 Water content

**Fig 6:**
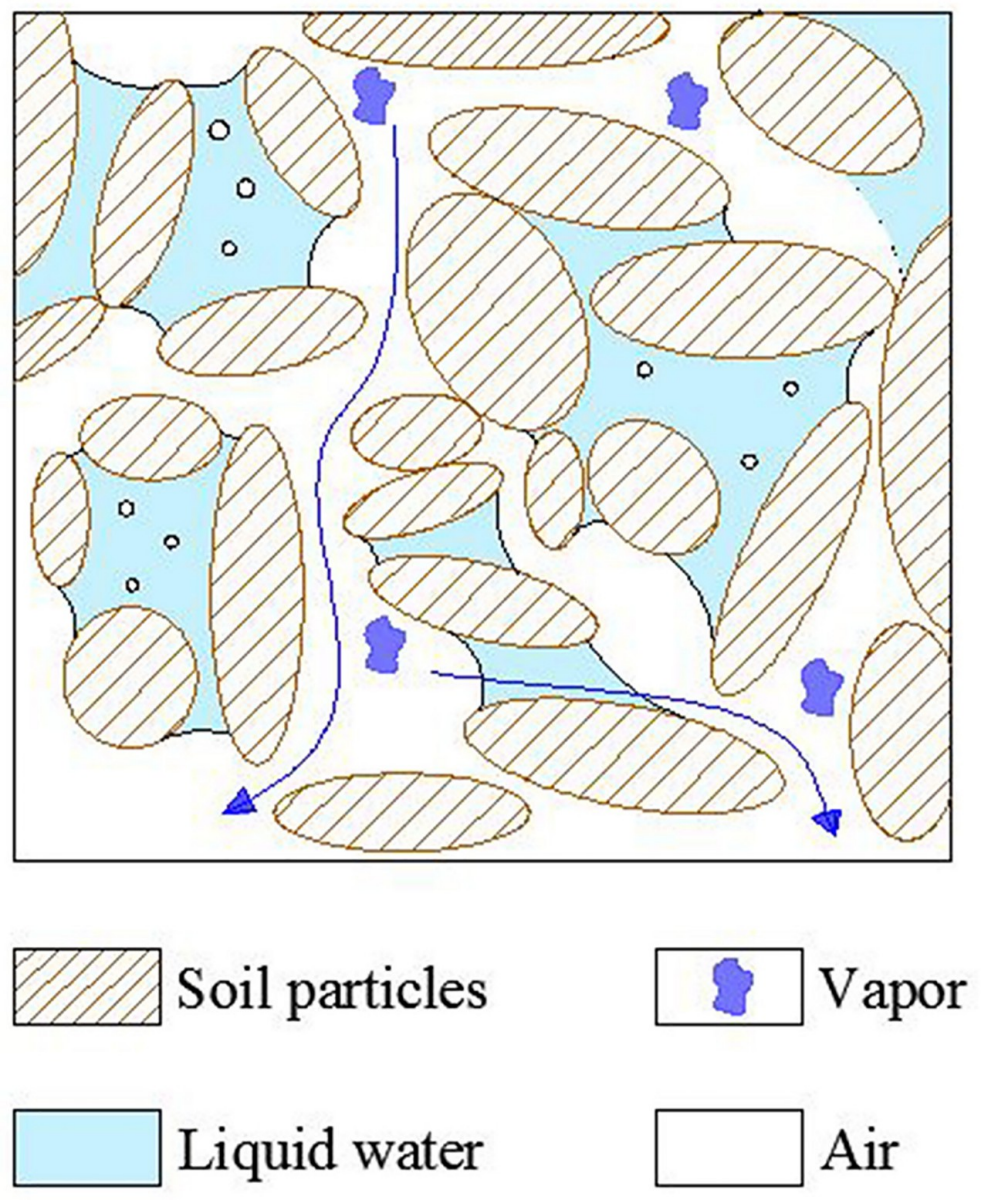
Schematic diagram of the initial state of the soil column.

**Fig 7:**
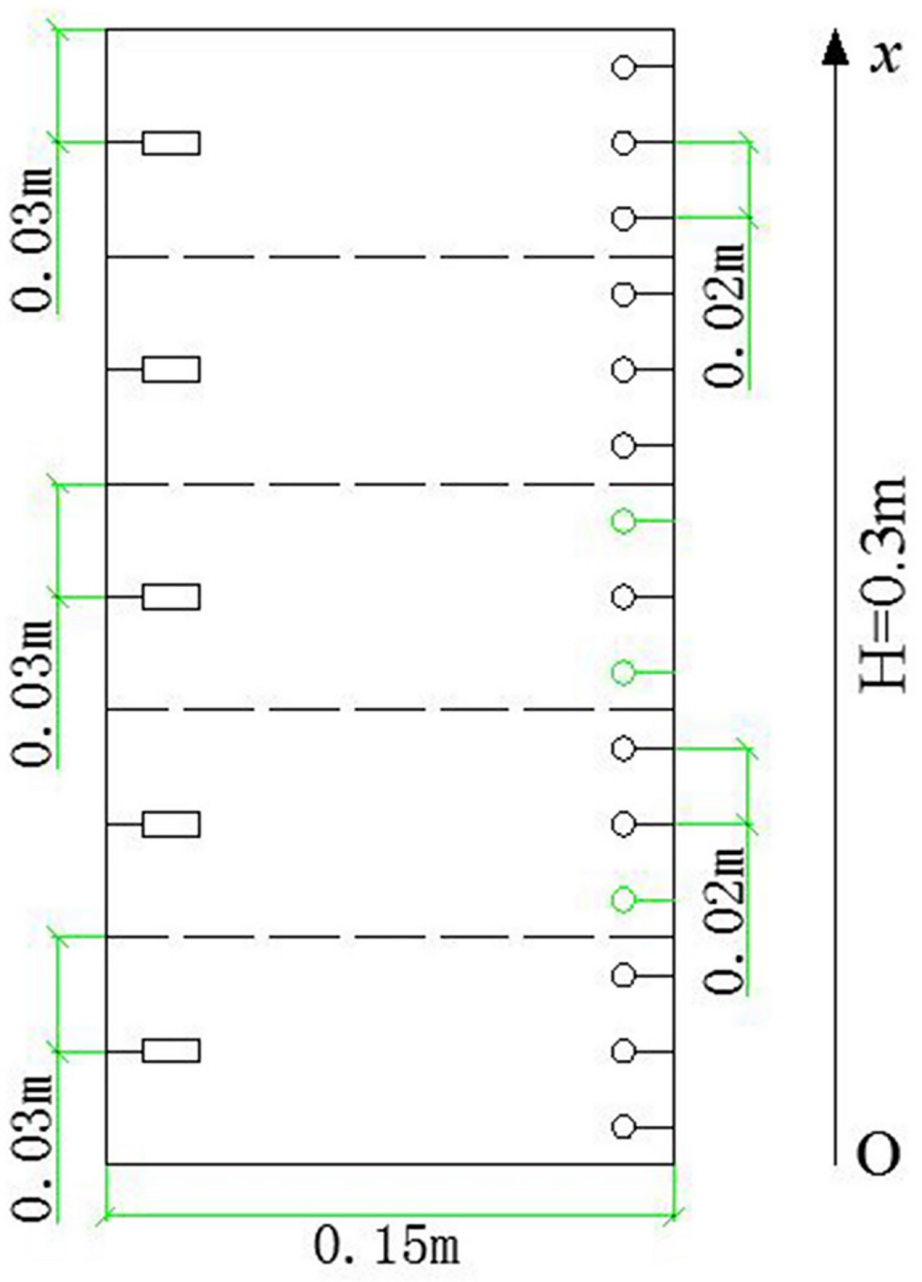
Schematic diagram of soil column division and the sensor distribution. (The rectangle represents the moisture sensor, and the circle represents the temperature sensor).

Fig 6 is the schematic diagram of the initial state of the soil column. Based on the initial water content and saturation of the soil column tested in this paper, it is concluded that the unsaturated soil consists of soil particles, air, water vapor and liquid water, and the initial state is a double open system with air and water connected [14]. Wherein, the contact forms of soil particles are direct contact, meniscus connection and non-contact. As shown in Fig 6, air exists in the pores between soil particles or in liquid water, while water vapor exists in the air. Meanwhile, liquid water exists in the pores between soil particles in the form of meniscus water. When water migration occurs, water can move both in the pore space and in the meniscus water, as shown by the arrows in Fig 6. Suppose the whole area of the soil column is divided into five equal parts each associated with a volumetric water content sensor which is positioned in the center of the corresponding part. Fig 7 shows the schematic diagram of the soil column division and the sensor distribution. Thus, the data obtained by the moisture sensors at the distance of 0.03m, 0.15m and 0.27m from the bottom of the soil column are reasonably assumed to represent the water content variations of the top, middle and bottom part.

**Fig 8:**
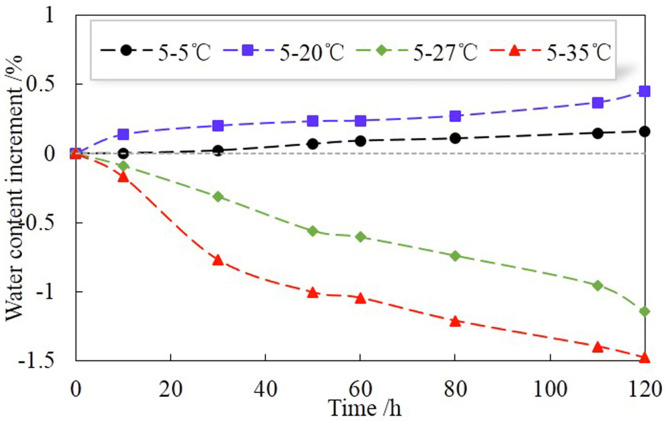
Variations of volumetric water content (Δ*θ*) at the bottom of H for different temperature gradients.

Fig 8 shows the volumetric water content (Δ*θ*) variations at the bottom of H for different temperature gradients. Existing studies [14] have shown that under the action of temperature gradient, the water in soil column migrates from the high-temperature end to the low-temperature end, and the water content at the bottom will increase under the closed system. However, the boundary condition at the bottom of the soil column in this study is free drainage, so the water content at the bottom soil increases when the temperature gradient is low, and decreases when the temperature gradient is high. This indicates that the higher the temperature gradient is, the more energy the water absorbs. When the absorbed energy exceeds a certain amount, the water in the unsaturated soil can overcome the capillary suction between soil particles and become free water to discharge. In addition, as can be seen, the higher the temperature gradient, the greater the amount of water migration. This can be explained from the following reasons: the migrated water contains liquid water and water vapor. The increase in temperature makes the viscosity coefficient of liquid water decrease, while the pressure difference between the two sides of the meniscus increases, causing the liquid water a better flow performance and an increased migration amount. The vapor density is a function of temperature, and an increase in temperature increases the density of water vapor, resulting in more vapor water participating in moisture migration. Moreover, the expansion or release of air by heating will also promote the migration of moisture.

**Fig 9:**
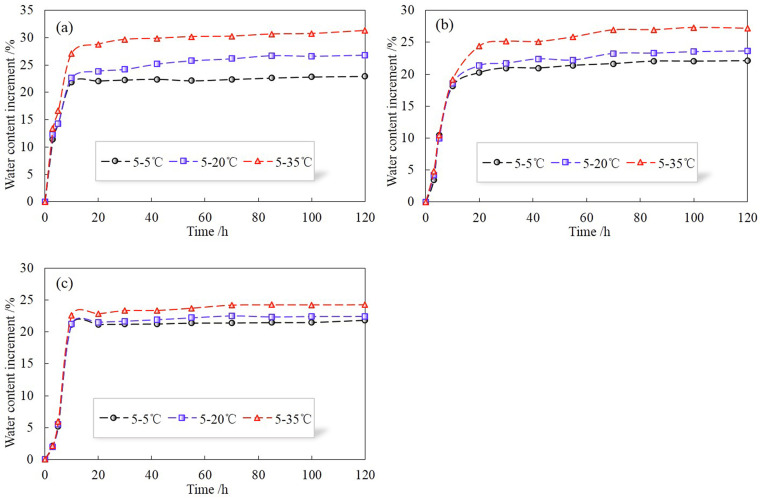
Variations of volumetric water contents (Δ*θ*) at different positions of HI for different temperature gradients. (a) top part, (b) middle part, (c) bottom part.

Fig 9 shows the variations of volumetric water contents (Δ*θ*) at different positions of HI for different temperature gradients. When the soil column is at constant temperature, as rainwater enters the soil column, the water content at each location increases first and then remains unchanged. The closer to the top region, the earlier the moisture field reaches a steady state. Besides, there is little difference in water content at different locations after the water content does not change. Comparing with the case of constant temperature, the water content at each position increases when there is a temperature gradient in the soil column. And the higher the temperature gradient at the same position, the greater the increment of water content. The reasons are explained as follows: (1) similar to H, the existence of temperature gradient will lead to water migration inside the soil column, which provides more space for external water to enter the soil; (2) when the water with temperature enters the soil column, the scour and lubrication effect between soil particles is enhanced, causing some soil particles to slip and stagger and more pores are generated in the soil column, and the higher the temperature is, the more obvious the effect will be; (3) under the effect of long-term temperature, part of soil particles in the soil skeleton are separated from each other and suspended in the pores, which increases the specific surface area of soil particles and enables them to absorb more water; (4) the thermal expansion caused by the increase in temperature facilitates the discharge of air, which also provides more space for water to fill the soil pores. In addition, it can be found that the increment of water content is greater near the top soil when the temperature gradient exists. This is because part of the fine soil particles are washed to the lower part, resulting in greater porosity in the upper part of the soil column; at the same time, the average temperature level in the upper part of the soil column is higher than that in the lower part, so the moisture increase caused by temperature is more obvious near the warm end. In the test, it was also observed that the outflow water became more turbid as the temperature gradient increased, indicating that more fine particles were washed and flowed out of the soil.

**Fig 10:**
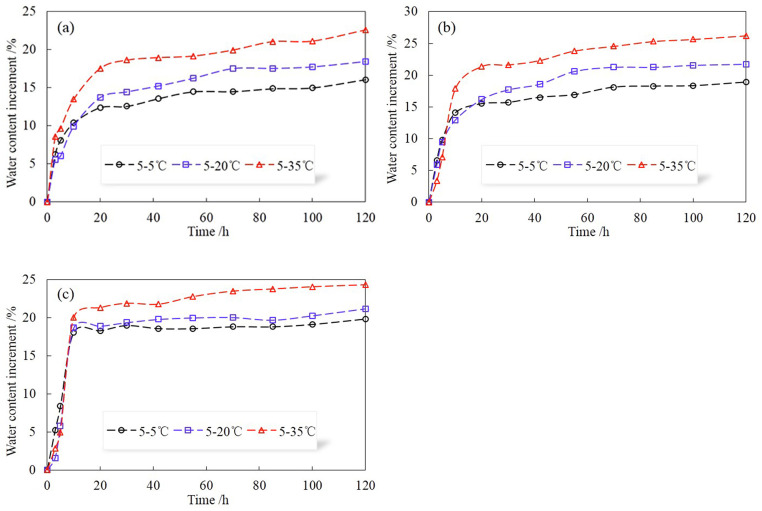
Variations of volumetric water contents (Δ*θ*) at different positions of P for different temperature gradients. (a) top part, (b) middle part, (c) bottom part.

**Fig 11:**
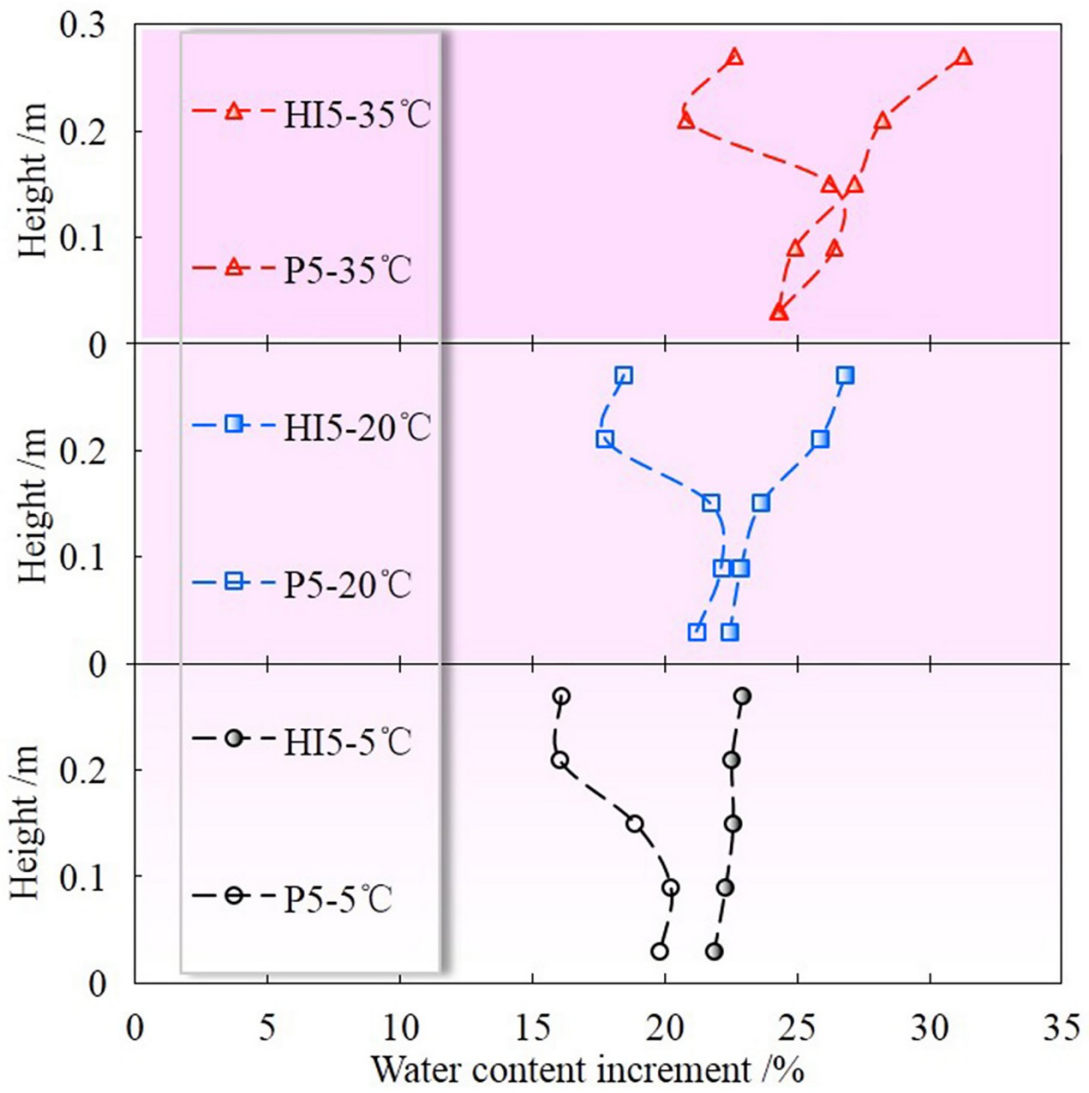
Water distribution of HI and P at 120h at different temperature gradients.

Fig 10 shows the variations of volumetric water contents (Δ*θ*) at different positions of P for different temperature gradients. As can be seen, the water content at each location increases with the temperature gradient, which is consistent with HI. However, the increment of water content of P at the same position is less than HI. This is because the water in HI migrates layer by layer from top to bottom and causes the entire soil column to reach saturation, but the preferential flow phenomenon caused by the macropore structure keeps the soil column unsaturated. Besides, the water content of P still increases slowly after the water outflow, especially in the soil matrix with macropore structures. This is due to the fact that the water in P preferentially passes through the macropore region and then diffused around, so some pores that have not been wetted will be gradually filled with water until the water content of the soil column no longer changes. As shown in Fig 11, the water content near the end of the preferential flow channel is much larger than that in other parts, indicating that the appearance of preferential flow results in uneven distribution of soil moisture in the vertical direction. Simultaneously, the increase in temperature gradient will intensify this phenomenon.

Table 3 shows the comparison of the outflow times of HI and P. As can be seen, the outflow time of P is earlier than that of HI at the same temperature gradient. However, whether there is preferential flow in the soil column or not, the outflow time is delayed with the increase of temperature gradient. This is also consistent with the conclusions drawn above that the water content in the soil column increases with the increase of temperature gradient whereas the existence of preferential flow will lead to different changes in the hydraulic field in the soil column.

**Table 3:** Outflow time of HI and P at different temperature gradients.

Temperature gradient	Outflow time/h	
	HI	P
5–5°C	7.9	6.85
5–20°C	8.2	7.15
5–35°C	8.5	7.7

### 3.2 Temperature

**Fig 12:**
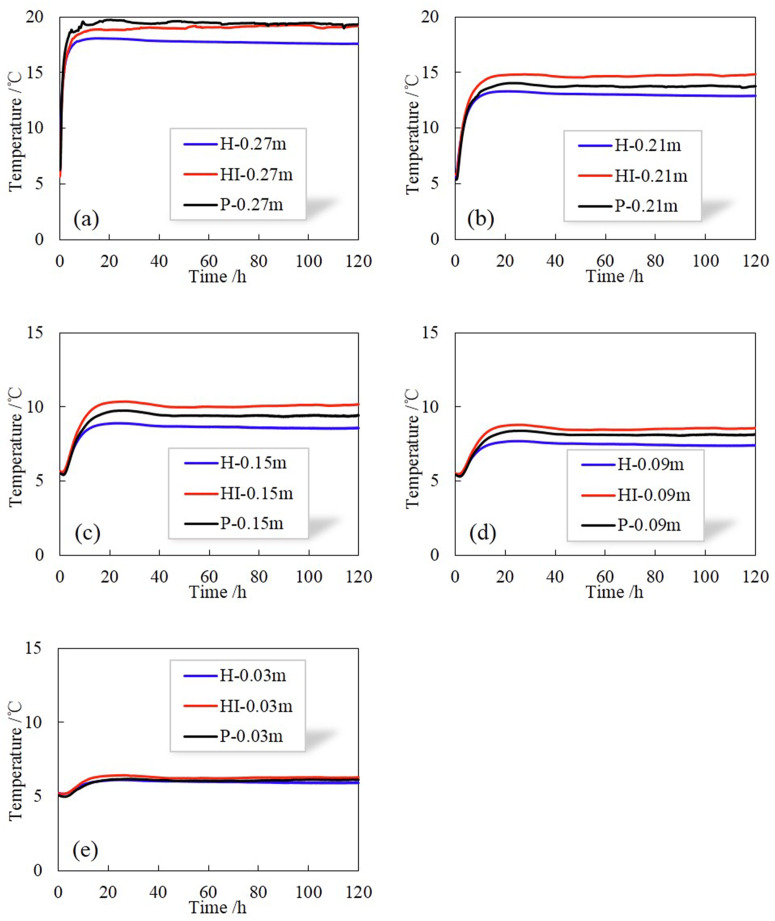
Variations of temperature at the same position in different soil columns at 5–20°C.

**Fig 13:**
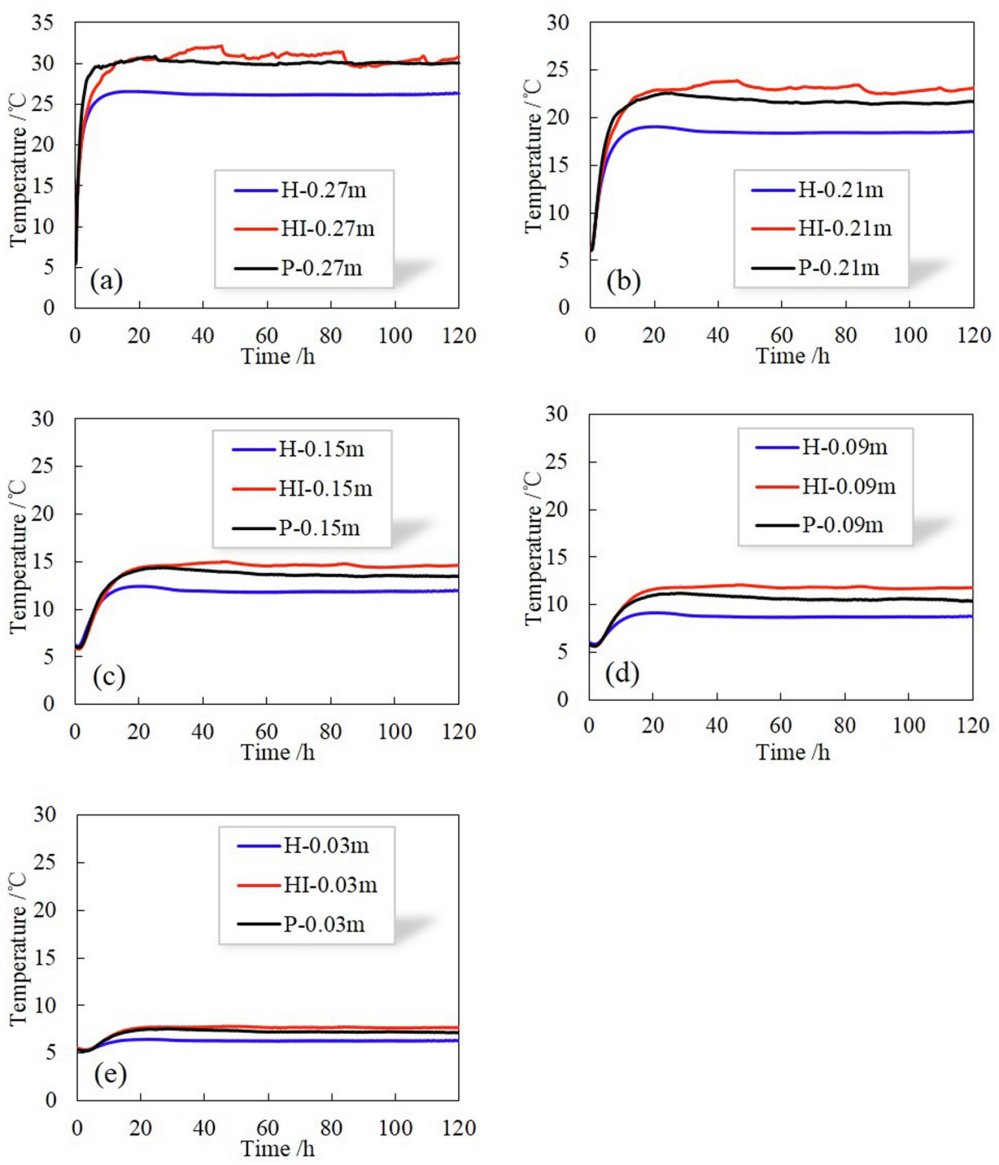
Variations of temperature at the same position in different soil columns at 5–35°C.

As shown in Fig 7, the data obtained by the temperature sensors in the center of each part of the soil column (at the distance of 0.03m, 0.09 m, 0.15m, 0.21m and 0.27m from the bottom of the soil column) are adopted to analyze the temperature variations during the test. Figs 12 and 13 illustrate the variations of temperature at the same position in different soil columns at 5–20°C and 5–35°C, respectively. When rainfall infiltration occurs, the temperature at all locations of the soil column increases compared to the case without infiltration. This is because the filling of soil pores by rain increases the area of heat conduction, and the heat carried by rainwater is transferred to the whole soil column along with the flow, so the heat transfer of soil column is enhanced and the temperature at each position rises. In addition, it can be observed that the closer to the warm end, the greater the increase in temperature, which is due to the shorter the path of heat transfer, the less heat loss.

However, the temperature changes of HI and P are different under infiltration conditions. In terms of the process of temperature increase: when the temperature at the top of the soil column is 20°C, the heating rate of P is faster than HI in the region above 0.27m height; while when the temperature at the top is 35°C, the heating rate of P is faster than HI in the region above 0.15m height, and reaches the steady state earlier. Therefore, it can be concluded that the preferential flow can accelerate the process of heat transfer in soil within a certain depth range, resulting in a shorter time for the temperature to reach steady state. Meanwhile, the larger the temperature gradient is, the larger the area where heat transfer is accelerated.

**Fig 14:**
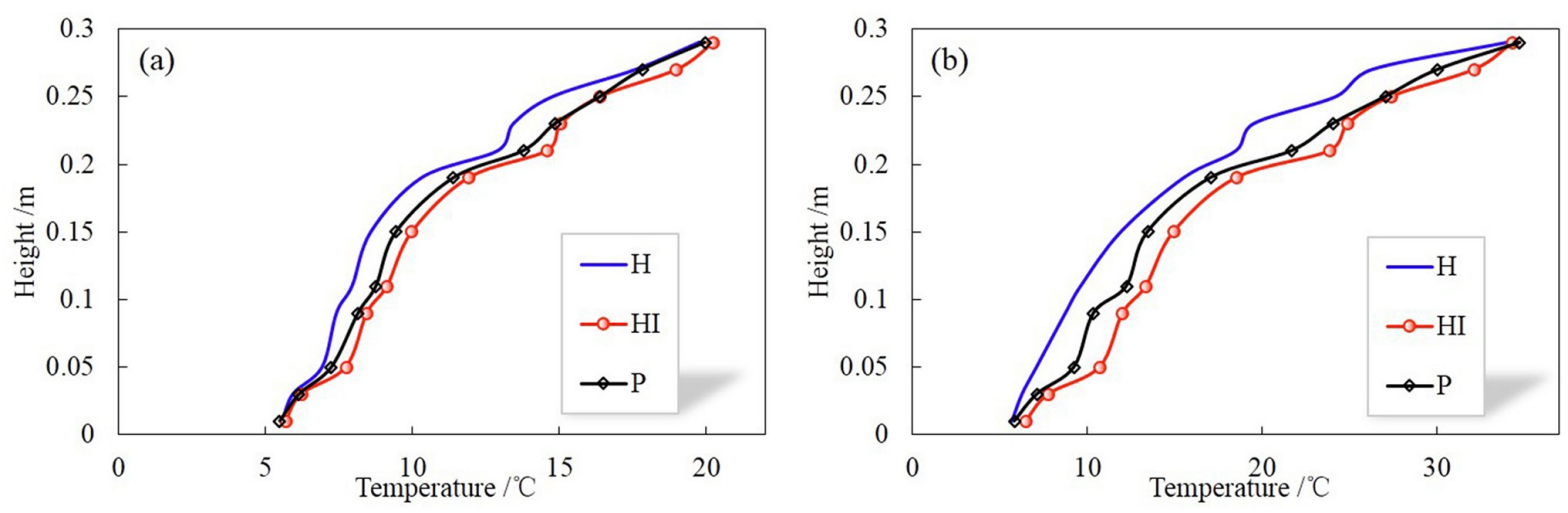
Temperature distribution of H, HI and P in different temperature gradients at 120 h. (a) 5–20°C, (b) 5–35°C.

In terms of the result of temperature increase: the temperature at the steady state of P is lower than HI. Combined with the results of water content above, that is, the distribution of water content in HI is greater than P, demonstrating that the main factor affecting the steady-state temperature is the moisture content, the higher the water content, the higher the temperature at steady state. Simultaneously, the larger the temperature gradient, the larger the temperature difference ΔT at the same location, which can also be seen from the distribution of temperature along the soil column at 120 h shown in Fig 14.

### 3.3 Displacement

**Fig 15:**
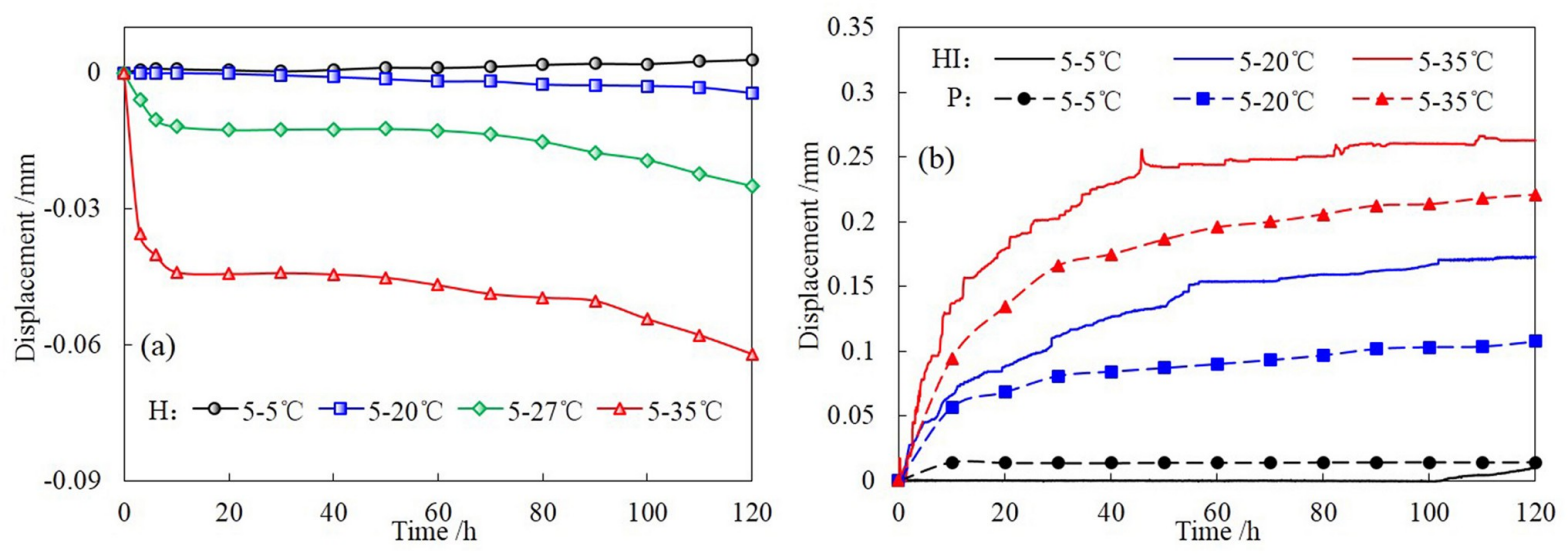
Variations of top displacement of different soil columns for different temperature gradients. (a) H, (b) HI, P.

Fig 15(a) illustrates the variations of top displacement of H for different temperature gradients. As can be seen, soil settlement occurs under the effect of temperature gradient, and the amount of settlement increases with the increase of temperature gradient. This is because under the action of temperature gradient, the water in the soil column migrates downward and drains out, then the thickness of the water film between soil particles decreases, which leads to the enhancement of the intermolecular force and capillary force. Consequently, the soil particles are close to each other and the soil pores are compressed, exhibiting the behavior of soil settlement. In addition, the larger the temperature gradient, the more obvious the soil settlement.

Fig 15(b) shows the changes in top displacement of HI and P for different temperature gradients. Similarly, the change of displacement increases with increasing temperature gradient. While different from the result of H, the displacement changes of HI and P are characterized by expansion. The reasons are explained as follows: Firstly, after the infiltrating water enters the soil column, the adsorption and capillary forces in the soil gradually weaken as the water content within the soil column increases, which will lead to the weakening or disappearance of the tendency of soil particles approaching each other. Secondly, under the action of seepage force, soil particles will slip and stagger, and more pores are interconnected and filled with water, thus causing expansion; Thirdly, infiltration water with the same temperature as the boundary of the soil column continuously enters the soil column, leading to thermal expansion effects. Furthermore, it can be found that the expansion of HI is greater than P at the same temperature gradient, which is related to the greater moisture content in HI.

### 3.4 Interaction between different fields

The thermo-hydro-mechanical variations of unsaturated soils during different experimental conditions are presented and analyzed in this paper, from which we can see the interaction between thermal field, hydraulic field and mechanical field.

The effect of the thermal field on the hydraulic field is reflected in the migration of water from the warm end to the cold end due to the increase in soil water potential at the warm end of the soil column without infiltration. While in the case of infiltration, the existence of temperature gradient increases the pore channels and the specific surface area of fine particles, which leads to the increase of water content in the soil column and the delay of outflow time. Due to the limitation of testing means, only the deformation results in the vertical direction of soil column are used to reflect the changes of mechanical field in this paper. The temperature field affects the mechanical field, which is reflected by the fact that the existence of temperature gradient causes the soil column to settle without infiltration and expand when infiltration occurs. Besides, the larger the temperature gradient, the more obvious the hydraulic-mechanical response induced by thermal.

The hydraulic field has influence on both the process of temperature field formation and the final distribution. For example, faster water migration in a preferential flow soil column results in faster heat transfer and earlier arrival at the steady state. And when the temperature does not change, the soil column with higher water content has higher steady-state temperature due to better thermal conductivity characteristics. Based on the experimental results in this paper, it can be found that the effect of water change on deformation is closely related to the presence of temperature field. The temperature gradient affects the water distribution, and the coupling of heat and water transfer leads to the deformation of the soil column.

The top displacement of the soil column measured in this paper partially reflects the response of the mechanical field to thermo-hydraulic variations, i.e., the coupling effect of thermo-hydro results in the settlement or expansion of the soil. However, due to the small amount of deformation measured in the test, the influence of mechanical field on thermal and hydraulic fields cannot be well reflected based on the test results in this paper. Subsequently, we can consider adding suction sensors to test other mechanical properties to improve the analysis of the coupling mechanism.

## 4. Conclusion

Rainfall infiltration is an important factor inducing landslides in soil slopes, and the presence of temperature gradients in summer rainfall slope soil can cause water migration and affect the thermal-hydraulic-mechanical characteristics of the soil. Therefore, it is necessary to carry out the corresponding model test research. In this paper, an experimental device have been developed that can test the temperature, moisture content and displacement changes of unsaturated soil columns under the combined effect of temperature gradient and rainfall infiltration. Using the test apparatus, the model tests of homogeneous soil column (H), homogeneous soil column with infiltration (HI), and preferential flow soil column with infiltration (P) under different temperature gradients are respectively conducted. No runoff is generated during infiltration, and the temperature of infiltration rainwater is consistent with that of the warm end. The preferred flow path is a semi-through form that connected to the top of the soil column, whereas the bottom of the soil column drains freely. Through the comparative analysis of the THM changes during the test, the following conclusions are summarized:

When rainfall infiltration occurs, the temperature of the soil column will increase due to the heat carried by the rainwater. Under rainfall conditions, the macropore structure leads to a faster heat transfer in the soil, resulting in a shorter time for the temperature to reach steady state. While the vertical temperature distribution is less than that of the homogeneous soil column after the soil temperature changes stabilized. In addition, the above phenomenon will be more obvious with the increase of temperature gradient.

When rainfall infiltration occurs, water within homogeneous soil column gradually migrates downward from the top to the bottom. However, for the preferential flow column, the water first migrates downward through the macropore structure and then diffuses around. When the water content remains almost unchanged, the water content near the end of the preferential flow channel is much larger than that in other parts. In addition, compared with the homogeneous soils, the outflow time of the preferential soil column is earlier, but the time of water field reaching steady state is later.

Only when the temperature gradient exits, the displacement of the soil column changes, which is shown as settlement without infiltration and expansion with infiltration. Despite the small amount of displacement variation, it can still be seen that the settlement and expansion show an increasing trend with the increase of temperature gradient. Therefore, if the effect of temperature gradient is ignored, it may cause some deviations in the study of some special soils (e.g., collapsible loess and bentonite) in hot and rainy areas, and relevant studies will be carried out for discussion subsequently.
